# TactiGraph: An Asynchronous Graph Neural Network for Contact Angle Prediction Using Neuromorphic Vision-Based Tactile Sensing

**DOI:** 10.3390/s23146451

**Published:** 2023-07-17

**Authors:** Hussain Sajwani, Abdulla Ayyad, Yusra Alkendi, Mohamad Halwani, Yusra Abdulrahman, Abdulqader Abusafieh, Yahya Zweiri

**Affiliations:** 1 UAE National Service & Reserve Authority, Abu Dhabi, United Arab Emirates; hussainmsajwani@gmail.com; 2 Advanced Research and Innovation Center (ARIC), Khalifa University, Abu Dhabi 127788, United Arab Emirates; abdulla.ayyad@ku.ac.ae (A.A.); yahya.zweiri@ku.ac.ae (Y.Z.); 3 Department of Aerospace Engineering, Khalifa University, Abu Dhabi 127788, United Arab Emirates; 4 Research and Development, Strata Manufacturing PJSC, Al Ain 86519, United Arab Emirates

**Keywords:** tactile sensing, vision-based tactile sensing, event-based vision, robotic manufacturing

## Abstract

Vision-based tactile sensors (VBTSs) have become the de facto method for giving robots the ability to obtain tactile feedback from their environment. Unlike other solutions to tactile sensing, VBTSs offer high spatial resolution feedback without compromising on instrumentation costs or incurring additional maintenance expenses. However, conventional cameras used in VBTS have a fixed update rate and output redundant data, leading to computational overhead.In this work, we present a neuromorphic vision-based tactile sensor (N-VBTS) that employs observations from an event-based camera for contact angle prediction. In particular, we design and develop a novel graph neural network, dubbed TactiGraph, that asynchronously operates on graphs constructed from raw N-VBTS streams exploiting their spatiotemporal correlations to perform predictions. Although conventional VBTSs use an internal illumination source, TactiGraph is reported to perform efficiently in both scenarios (with and without an internal illumination source) thus further reducing instrumentation costs. Rigorous experimental results revealed that TactiGraph achieved a mean absolute error of $0.62^{\circ }$ in predicting the contact angle and was faster and more efficient than both conventional VBTS and other N-VBTS, with lower instrumentation costs. Specifically, N-VBTS requires only 5.5% of the computing time needed by VBTS when both are tested on the same scenario.

## 1. Introduction

### 1.1. Sense of Touch and Vision-Based Tactile Sensing

The sense of touch is an important feedback modality that allows humans to perform many tasks. Consider, for example, the task of inserting a key into a lock. After obtaining an estimate of the keyhole’s position, we rely almost exclusively on the sense of touch to move the key from being in the general vicinity of the keyhole to inserting it in the keyhole. During these fleeting seconds, we rely on tactile feedback to adjust both the position and the orientation of the key until insertion is obtained. More subtly, we also use tactile feedback to adjust how much force is needed to keep the grasped key from slipping. For robots to perform tasks such as grasping [1,2], peg-in-hole insertion [3,4], and other tasks that require dexterity, it becomes paramount that robotic systems have a sense of touch [5,6,7]. Much work has been conducted on augmenting robots with an artificial sense of touch [8]. Several tactile sensor conceptions exist within the literature. These include sensors based on transduction (capacitive, resistive, ferromagnetic, optical, etc.) as well as those based on piezoelectric material [7]. However, these sensors have high instrumentation costs and are thus hard to maintain over long periods of time [9]. A particularly promising tactile sensing technology is vision-based tactile sensors (VBTSs) [9].

VBTS systems consist of an imaging device capturing the deformation of a soft material due to contact with the external environment. The soft material is equipped with a pattern that allows the image sensor to capture deformations clearly. Such patterns include small colored markers [10,11], randomly dispersed fluorescent markers [12], and colored LED patterns [13]. Compared to other tactile sensors, VBTSs do not require much instrumentation; only the imaging device and a source of illumination are required to be instrumented and maintained. This is important for the longevity and usability of the sensor. Although VBTSs have low instrumentation overhead, they provide high-resolution tactile feedback. VBTS output images, and thus can be processed using classical and learning methods for image processing. Some algorithms build on intermediate features such as marker position, optical flow, or depth [3,10,11,14,15,16,17,18], while others build and train end-to-end models [3,18,19,20]. The high resolution of VBTS allows robots to perform tasks such as detecting slip [2,21,22], estimating force distribution [11,23], classifying surface textures [24,25], and manipulating small and soft objects [26,27] as well as many tasks that require dexterity and fine-detailed pose estimation.

**Figure 1:**
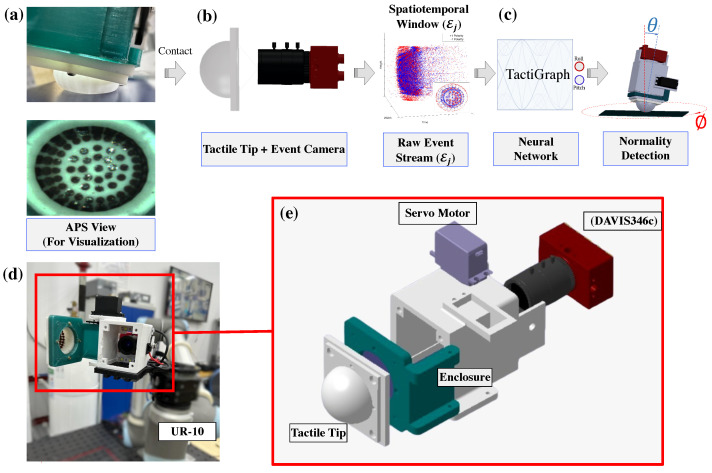
Proposed framework. (**a**) The neuromorphic vision-based tactile sensor makes contact with the surface causing the tip of the sensor to deform. Markers placed on the inner surface of the tip are displaced due to the deformation of the sensor. This can be seen in the active-pixel sensor (APS) view. This is shown for visualization only and is not used in any further processing. (**b**) The displacement of the markers is captured by an event-based camera, hence generating a continuous stream of events. A volume of events $E_{j}$ is used to construct a graph $G_{j}=\left(V_{j},E_{j}\right)$. (**c**) This graph is processed by TactiGraph, a graph neural network that predicts the contact angle of the sensor. (**d**) The full sensor with the hatch open. The markers on the inner wall of the tactile tip can be seen. (**e**) The proposed sensor configuration comprises four main parts: the deformable tactile tip, an event-based camera, a single servo motor to open and close the hatch, and an enclosure holding the camera.

In our previous work [10,28], we have introduced a multifunctional sensor for navigation and guidance, precision machining, and vision-based tactile sensing. Concretely, the sensor is introduced in the context of the aerospace manufacturing industry, where maintaining high precision while machining is imperative. The proposed sensor configuration is depicted in Figure 1. When the hatch is open by the servo motor, the camera used for VBTS is used to navigate to the desired position. Once the target position is achieved, the hatch is closed, and the deburring or drilling process starts. The tactile feedback from the VBTS is used to ensure that the robot is machining while maintaining perfect perpendicularity to the workpiece. The video stream of the imaging device is processed by a convolutional neural network (CNN). Ensuring perpendicularity is crucial when working in the aerospace manufacturing industry [29,30,31]. Failure to abide by perpendicularity requirements leads to an increase in bending stress and a decrease in fatigue life, thus lowering the reliability of aircraft parts [32,33]. Several other works in the literature use VBTS for contact angle prediction [19,34,35]. Lepora et al. [19] use a CNN on binarized images from a camera to estimate the contact pose. Psomopoulou et al. [34] also use a CNN to estimate the contact angle while grasping an object. Finally, Ref. [35] extract the markers’ positions using blob detection, then construct a 2D graph with markers as nodes using Delaunay triangulation. The graphs are then processed using a graph neural network to predict the contact angle. All of the aforementioned work uses conventional VBTS, thus requiring internal illumination as well as having a limited temporal update-rate.

Most VBTSs, including [10,19,28,34,35], rely on standard frame-based cameras. These cameras capture full-resolution frames consisting of pixel intensities at a fixed synchronous rate. Consequently, even when there are no changes in the scene, the frame-based cameras continue to capture frames, leading to redundant processing of unchanged pixels between consecutive frames. This redundancy does not contribute additional knowledge for downstream tasks. Moreover, the synchronous nature of frame-based cameras poses challenges when operating in scenarios that require fast perception and action. The latency due to the exposure time of synchronous cameras implies a delay in the action of the robot. For instance, in drilling and deburring tasks, it was essential to swiftly perceive unexpected events, such as a deviation from perpendicularity, and react promptly to prevent damage to the machine and workpiece [36,37]. High-speed cameras can reduce the latency due to exposure time; however, they result in more data that requires higher bandwidth to transfer, and more processing power, which ultimately incurs latency. Thus, in VBTS utilizing framed-based cameras, a compromise between exposure time latency and processing latency exists. Therefore, an asynchronous, low-bandwidth, and fast update-rate sensor is needed. The emergence of neuromorphic event-based cameras, driven by advancements in vision sensor technologies, has addressed these limitations and has become a critical tool for achieving accurate visual perception and navigation.

The event-based camera, a bio-inspired device, offers unprecedented capabilities compared to standard cameras, including its asynchronous nature, high temporal resolution, high dynamic range, and low power consumption. Therefore, by utilizing an event camera instead of a standard camera, we can enhance the potential of our previous sensor and improve navigation or machining performance in challenging illumination scenarios. In this work, we use an event-based camera for VBTS. This allows us to obtain less expensive computations, fast update rate, and relinquish the need for internal illumination, which adds instrumentation complexity.

### 1.2. Neuromorphic Vision-Based Tactile Sensing

Neuromorphic cameras (also known as event-based cameras) are a relatively new technology, first introduced in [38], that aim to mimic how the human eye works. Neuromorphic cameras report intensity changes, at the pixel level, in the scene in an asynchronous manner, rather than report the whole frame at a fixed rate. This mode of operation makes event-based cameras exhibit no motion blur. The pixel-wise intensity changes, called events or spikes, are recorded at a temporal resolution on the order of microseconds. Event-based cameras have been applied in autonomous drone racing [39], space imaging [40], space exploration [41], automated drilling [42], and visual servoing [43,44]. Neuromorphic cameras’ fast update rate, along with their high dynamic range (140 dB compared to conventional cameras with 60 dB [45]) and low power consumption, make them apt for robotics tasks [46]. Therefore, several studies have proposed the use of neuromorphic event-based cameras for vision-based tactile sensing (VBTS) [1,16,20,21,47,48,49,50]. In particular, event-based cameras are capable of providing adequate visual information in challenging lighting conditions without requiring an additional light source, owing to their high dynamic range. Due to not needing a source of illumination, a VBTS system that utilizes an event-based camera will have a lower instrumentation cost and thus require less maintenance in the long run. Specifically, the instrumentation cost and complexity of the tactile sensor include the cables, powering circuit, maintenance, and replacement of defective parts over the sensor’s lifetime. Such tactile sensor configuration utilizing an event-based camera would reduce instrumentation complexity, such as having fewer cables and a smaller power circuit, and hence require less maintenance and replacement of defective parts. While some VBTSs use a semitransparent, transparent, or translucent tactile surface to overcome the need for a source of illumination [9,13,51,52], this will make training end-to-end machine learning models difficult as the camera will capture extraneous information from the environment making it dependent on the object the sensor is contacting and the environment, thus limiting generalization. Event-based cameras allow us to overcome the instrumentation and maintenance costs of having a source of illumination while still maintaining the potential for training end-to-end models. As it currently stands, event-based cameras are a new technology which are still not in mass production, making the price of available cameras in the order of thousands of dollars. However, as event cameras gain prominence and enter mass production, the price is expected to decrease significantly over the next five years [46]. This is exemplified in the consumer-grade mass-produced event-based camera by Samsung, which sells for USD 100 [46,53], a price comparable to conventional cameras. These features of event-based cameras make them an attractive choice for VBTS. However, dealing with event-based data still poses a challenge, as will be discussed in the following subsection.

### 1.3. Challenges in Event-Based Vision and Existing Solutions

The temporally dense, spatially sparse, and asynchronous nature of event-based streams pose a challenge to traditional methods of processing frame-based streams. Early work on neuromorphic vision-based tactile sensing (N-VBTS) constructs images from event streams by accumulating events over a period of time and applying image-processing techniques. Such approaches are called event-frame methods. These approaches usually use synchronous algorithms and apply them over constructed frames sequentially; thus, event-frame approaches do not exploit the temporal density and spatial sparsity of event streams. For instance, Amin et al. [47] detect the incipient slip of a grasped object by applying morphological operations over event-frames and monitoring blobs in the resulting frame. This approach is not asynchronous and does not generalize well to tasks beyond slip detection. Ward-Cherrier et al. [16] construct encodings relevant to the markers’ position of the tactile sensors and then use a classifier to detect the object’s texture in contact. Their algorithm iteratively updates marker positions using events generated around markers. This method is synchronous and is susceptible to high noise, especially when there is no illumination. Furthermore, if there is a lot of motion, the estimated marker positions drift away from the actual marker positions. Fariborz et al. [48,49] use Conv-LSTMs on event-frames constructed from event streams to estimate contact forces. Faris et al. [22] uses CNN over accumulated event heatmaps to detect slip. This approach is not asynchronous and hence has downtime between constructed event-frames. To our knowledge, the only asynchronous deep learning method that makes use of spatial sparsity and temporal density applied in the N-VBTS setting is the work of MacDonald et al. [50].

Spiking neural networks (SNNs) are computational models inspired by the brain’s neural processes. They utilize event- or clock-driven signals to update neuron nodes based on specific parameters, using discrete spike trains instead of continuous decimal values for information transfer [54]. This biologically-inspired approach offers a more intuitive and simpler inference and model training compared to traditional networks [55]. Building on [16]’s NeuroTac, they propose using an SNN to determine the orientation of contact with an edge. While this is a promising step towards neuromorphic tactile sensing, SNNs are trained in an unsupervised manner. Another classifier is run on top of the SNN to make predictions. However, this approach does not generalize well beyond simple tasks. Furthermore, training SNNs is still challenging due to their non-differentiable nature and their requiring larger amounts of data for effective training due to the sparsity of spike events. This limitation can restrict their usability in domains with limited data availability. Additionally, SNNs require neuromorphic computing hardware for effective event-based processing [56,57].

Outside the N-VBTS literature, event-frame and voxel methods also persist [45,58,59,60,61]. An emerging line of research investigates the use of graph neural networks (GNNs) to process event streams [62,63,64,65,66]. GNNs operate on graphs by learning a representation that takes into account the graph’s connectivity. This representation can be used for further processing via classical machine and deep learning methods. GNNs generalize convolutional networks for irregular grids and networks [67]. By constructing a graph over events from an event-based camera, GNNs can perform spatially sparse and temporally dense convolutions. GNNs can also operate in an asynchronous mode by applying the methods proposed in [68,69] to match the nature of event-based streams. This mode of operation ensures that calculations only occur when there are events, as opposed to event-frame methods. The earliest work utilizing GNNs for event streams, [62], investigates object classification on neuromorphic versions of popular datasets such as Caltech101 and MNIST. Other works also tackle object detection and localization [62,63,68]. Alkendi et al. [66] use a GNN fed into a transformer for event stream denoising. Furthermore, [70] shows that GNNs work well in object detection tasks while performing considerably fewer floating point operations per event compared to CNNs operating on event-frames.

Graphs inherently do not encode geometric information pertaining to their nodes. They only encode information concerning the topological relationships between the nodes as well as the node features. Accordingly, constructing useful and meaningful representations of event data requires more than just the topological structure of a graph. Thus, it becomes imperative to choose an appropriate message-passing algorithm that encapsulates the geometry of events for exploiting the spatiotemporal correlations between events. Several graph geometric deep learning methods have been applied to event-based data in the literature. These include mixture model network (MoNet), graph convolutional networks (GCN), SplineConv, voxel graph CNNs, and EventConv [62,63,64,65,66]. The capability of SplineConv has been proved to operate asynchronously on event streams as proposed by [70]. Moreover, SplineConv has been shown to perform better and faster than MoNet as demonstrated in [64]. In addition, SplineConv has been verified to be more expressive than GCNs, which can only use one-dimensional features [71,72]. In the case of geometric graphs, this feature is usually taken as the distance between nodes. This is problematic for two reasons: (1) messages shared from two equidistant nodes will be indistinguishable and (2) the messages will be rotation invariant and will hence lose all information about orientation.

### 1.4. Contributions

In this work, we use a SplineConv-based graph neural network to predict the contact angle of a neuromorphic vision-based tactile sensor. This proposed framework is depicted in Figure 1. Our contributions can be summarized as follows.

- We introduce TactiGraph, a graph neural network based on SplineConv layers, which processes data from a neuromorphic vision-based tactile sensor. TactiGraph effectively handles the spatial sparsity and temporal density of event streams and is designed for solving the problem of contact angle prediction. TactiGraph achieves a mean absolute error of $0.63^{\circ }$ in predicting the contact angle.
- We devise a new augmentation technique that involves jittering events spatially in the scene. This technique enhances the robustness of the model against noise in the event data, particularly in situations when the illumination is low. We obtain an error of $0.71^{\circ }$ using this augmentation strategy when no illumination source is present.
- We rigorously demonstrate the robustness and validity of TactiGraph for the task of contact angle prediction. We show that TactiGraph is able to exploit the event-based nature of neuromorphic cameras.

### 1.5. Outline

The rest of this work is organized as follows. In Section 2, we discuss the data collection apparatus as well as the sensor design. In Section 3, we benchmark TactiGraph against other VBTS and N-VBTS methods. Finally, we conclude in Section 4.

## 2. Materials and Methods

In this section, we describe the experimental setup used to generate the data in this paper. Furthermore, we also describe the tactile sensor design, the sensor’s output format, as well as how to handle this output using TactiGraph.

### 2.1. Data Collection and Experimental Setup

The VBTS system consists of a camera, an enclosure containing the camera, and a hemispherical soft surface attached to the hatch, as seen in Figure 1. Small beads are placed on the insides of the soft tactile surface to generate contrast, allowing the camera to capture the deformation of the sensor with clarity. The event camera used is an IniVation DAVIS 346c with a resolution of 346 × 260 and a latency of 20 μs [73]. The sensor enclosure is made of 3D-printed ABS. Two LED strips are placed above and below the camera. More details on the manufacturing process of the elastomer surface and the enclosure can be found in [10,28]. The whole apparatus is attached to the Universal Robotics UR10 [74]. The UR10 pushes the tactile sensor against a flat surface at various angles of contact (A supplementary video is available at: <https://www.youtube.com/watch?v=OTUBglD0_kc>, accessed on 4 May 2023). This is shown in Figure 1. The contact angle is controlled by two parameters, θ∈Θ and ϕ∈Φ, where Θ = {0, 1, …, 9} and Φ is a collection of 20 angles around the circle. This variation in θ and ϕ can be seen in Figure 1. We collect 12 samples of each contact angle, thus ending up with a total of n = 1836 samples in total. The depth of each contact case is chosen randomly at a lengthbetween 5 mm and 15 mm from the tip of the sensor when relaxed. The randomness in contact depth ensures that our model can generalize to different contact profiles from light to heavy contact cases. To evaluate the performance of N-VBTS without internal illumination, this process is performed twice, once with the LED strips on and another time with the LED strips turned off.

To obtain the ground truth of the contact angle, intrinsic and extrinsic calibrations are performed. The event-based camera operates using the same optics principles used by conventional cameras. Thus, we can obtain the intrinsic camera parameters using conventional techniques. We use the ChAruco board and OpenCV on images constructed from frames to obtain the camera parameters. Once the camera parameters are found, the camera is attached to UR10, as seen in Figure 1d. Using the ChAruco board, the extrinsic calibration is performed using the exact same method as proposed in [28]. When the hatch is closed, the CAD model and the extrinsic parameters found earlier are used to obtain the measurements of the sensor with respect to the ChAruco board, as described in [28]. Using these measurements, the ground truth of the contact angle of the sensor is obtained. To convert events into frames to use for calibration, E2VID [45] is used offline. It is worth noting that the calibration parameters obtained using E2VID were found to be almost identical to those obtained using the active pixel sensor (APS) mode of the DAVIS 346c. It is also worth noting that using the described method of calibration, we obtain the accurate and precise positioning of the sensor’s tip. This ensures that when the sensor is used to adjust the angle, it will make contact with the workpiece and only modify its orientation without changing its position. In other words, the sensor does not move laterally when in contact. As long as the workpiece is securely fixed and the manipulator’s joints are not defective, there should be no shearing motion applied to the sensor during the adjustment process.

### 2.2. Preprocessing the Event Stream

**Figure 2:**
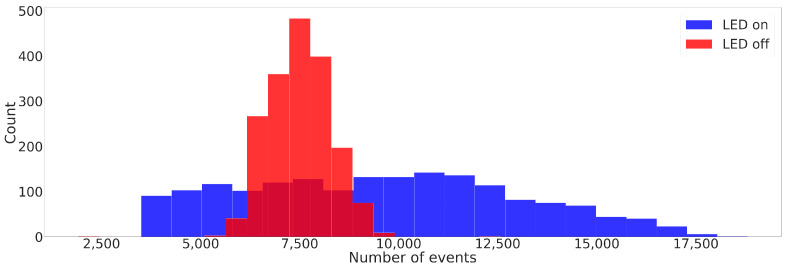
Histograms showing the number of events within each of the acquired event volumes ($E_{j}$) for different contact cases. The two histograms correspond to the two scenarios: LED-on and LED-off datasets. $E_{j}$ were acquired at specific contact angles (θ and ϕ). Furthermore, each contact case, represented by a pair of (θ, ϕ), was repeated 12 times at random depths.

Let C={1,…,240}×{1,…,346} be the set of all pixels in the DAVIS 346c. The output of the event camera is then a stream $S={\left\{e_{k}\right\}}_{k=1}^{N}$ of asynchronously reported intensity changes in the frame. The kth intensity change, an event, is a 4-tuple

(1) $$e_{k}=\left(x_{k},y_{k},t_{k},p_{k}\right)$$

where $(x_{k},y_{k})\in C$, $t_{k}\in R^{+}$, and $p_{k}\in \left\{-1,1\right\}.$ The (x,y) components represent where the event has happened, the *t* component is a timestamp indicating when the event has happened, and *p*, the polarity, represents whether the intensity change is positive or negative. For each event $e_{k}\in E_{j}$ we use a normalized spatiotemporal position $r_{k}=\left({\hat{x}}_{k},{\hat{y}}_{k},{\hat{t}}_{k}\right)=\left(\frac{x}{W},\frac{y}{H},\frac{t_{k}-t_{j}}{\Delta T_{j}}\right)$. Out of the stream S, for the jth contact case we capture a spatiotemporal volume $E_{j}\subset S$ such that

(2) $$E_{j}=\left\{\left(x,y,t,p\right)\in S\;|\;t_{j}\leq t\leq t_{j}+\Delta T_{j}\right\}$$

where $t_{j}$ the timestamp at which the jth contact case starts and $90\;\mathrm{ms}\leq \Delta T_{j}\leq 200\;\mathrm{ms}$ is the window size chosen to be, at most, 200ms. The window size is adaptive to adjust for various depths of contact cases; a heavy contact case takes more time than a light contact.Figure 2 shows histograms of the number of events generated by contact cases in both LED-on and LED-off scenarios. Specifically, for each contact case, the event volume ($E_{j}$) was acquired at the specified contact angle, assigned by θ and ϕ. Additionally, each contact case (represented by a pair of (θ, ϕ)) was repeated 12 times at random depths, as explained in Section 2.1. The variance in the depth of the contact translates itself into the variance of the number of events generated; a light contact case will cause a smaller displacement to the markers hence generating fewer events and vice versa. When comparing LED-on and LED-off histograms, it was observed that (I) on average, LED-on contact cases triggered more events than LED-off contact cases, and (II) the variance in the number of events for LED-off contact cases was lower than for LED-on contact cases. The reduced contrast between markers and the sensor when the LED is off results in fewer events generated by the camera. Additionally, dark scenes contribute to significant background noise [75,76]. While various denoising methods are proposed in the literature [66,75,76], we employ the background activity filter with a 1.25-ms time window when the LED strips are off.

### 2.3. Data Augmentation

To enhance the robustness of the model against noise, the dataset underwent an augmentation process prior to the training stage. Various augmentation techniques have been proposed in the literature for event-based streams. Some of these methods are inspired by traditional image-based augmentations, such as flipping, rotating, and mixing, among others [77,78]. Additionally, other strategies involve randomly shuffling events temporally between created event-frames [49] or randomly omitting events [79]. As we aim to predict contact angles, which are geometric measurements, methods that alter the scene’s geometry, such as flipping or rotating, are unsuitable as they would require adjusting the contact angle accordingly. Furthermore, even if we were to adjust the contact angle according to the augmentation strategy used, there is still an assumption of perfect symmetry in the fabrication of the sensor. This assumption, however, is not true. Thus, we avoid employing such geometric methods in our dataset. Furthermore, the proposed approach by [48] of shuffling events between event-frames is not suitable for our continuous time graphs. Therefore, we have developed a new method to enhance event-based streams by introducing the spatial jittering of events by a small amount. Specifically, given an event e=(x,y,t,p), we apply spatial jittering by at most one pixel, resulting in $\tilde{e}=\left(x+\delta x,y+\delta y,t,p\right)$ where δx and δy are uniformly drawn from the set {−1,0,1}. We will investigate the effects of this jittering technique in Section 3.1.1.

### 2.4. Graph Construction

For each spatiotemporal volume of events $E_{j}$ a graph $G_{j}=\left(V_{j},E_{j},X_{j}\right)$ is construced. The nodes of the graph are events themselves $V_{j}=E_{j}.$ The edges of the graph are determined using the kth nearest neighbors algorithm. The distance between two events $e_{i},e_{k}\in E_{j}$ is calculated using the Euclidean distance between their normalized spatiotemporal positions $r_{i}$ and $r_{k}.$ Letting $k\mathrm{NN}(e_{k})$ denote the set of the *k* nearest events to $e_{k}$, the set of edges of the graph is defined by

(3) $$E_{j}=\left\{\left(e_{i},e_{k}\right)\in E_{j}\times E_{j}\;|\;e_{k}\in k\mathrm{NN}\left(e_{i}\right)\right\}$$

While this method of constructing the graph does not always result in a graph with one connected component, we found it always results in one large connected component with a few smaller components consisting mostly of fewer than ten events. Thus, these smaller components are dropped out from the dataset. Finally, each node of the graph $e_{k}$ has the polarity $p_{k}$ as its node features, $X_{j}=\left\{p_{k}\;|\;e_{k}\in E_{j}\right\}$. The dataset is now the collection of the *n* graphs, with labels corresponding to the contact angle of the contact case collected from each of the *n* contact cases, $D_{\mathrm{LED}-\mathrm{on}}={\left\{\left(G_{j},{\mathrm{Roll}}_{j},{\mathrm{Pitch}}_{j}\right)\right\}}_{j=1}^{n}$. We note that we use a roll-pitch representation of the contact angle. This is to avoid singularities caused around 0. Another dataset, $D_{\mathrm{LED}-\mathrm{off}}$, with n=1836 samples is generated identically while the LED is off. The two datasets are used separately. Each dataset is randomly split into training, validation, and test subsets, with 70% of D being used for training while 15% is used for validation, and the last 15% is used for testing.

Message-passing is a mechanism used in GNNs to update node features in a graph. Given a graph *G* with nodes *V*, node features *X*, and edges *E*, a single layer of message passing will have nodes obtaining messages from their neighboring nodes and using those messages to update their node features. These messages are learnable functions of the neighboring nodes’ features. A particular type of message-passing is the convolutional type, where nodes update their representation in a way that resembles convolutions. A node u∈V updates its own representation from $x_{u}^{\ell }$ at layer *ℓ*, to $x_{u}^{\ell +1}$ at layer ℓ+1 by

(4) $$\begin{array}{cc} m_{uv} & =a_{uv}\varphi \left(x_{i}^{\ell },x_{j}^{\ell }\right) \end{array}$$

(5) $$\begin{array}{cc} m_{u} & =\sum_{v\in N(u)}m_{uv} \end{array}$$

(6) $$\begin{array}{cc} x_{u}^{\ell +1} & =\psi (x_{i}^{\ell },m_{i}) \end{array}$$

where v∈V is a neighboring node of *u*, $a_{uv}$ are edge attributes, and φ and ψ are learnable functions [67].

### 2.5. TactiGraph

**Figure 3:**
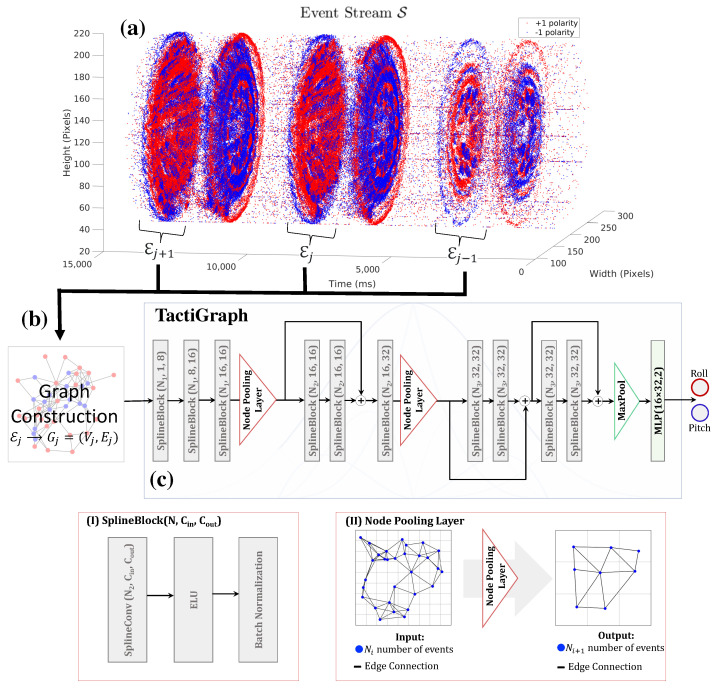
(**a**) The temporally dense and spatially sparse raw event stream S resulting from the data collection setup. The polarity of each event, represented by either positive (+1) or negative (−1), is indicated by blue and red colors, respectively, indicating the direction of the detected change in pixel intensity by the event camera. We see that the events generated cluster in large circles. Each one of the large circles in the stream corresponds to a compression or retraction of the tactile sensor. Inside these circles, traces of the markers’ movement can be seen. Spatiotemporal volumes $E_{j}$ corresponding to the large circles are extracted. Each volume has a temporal width $\Delta T_{j}$. (**b**) Out of each spatiotemporal volume $E_{j}$, a graph $G_{j}$ is constructed. (**c**) The graphs are fed into TactiGraph whose architecture is shown. The building blocks of TactiGraph are the (**I**) SplineBlock and the (**II**) node pooling layers. The SplineBlock consists of a SplineConv layer and an ELU activation layer, followed by a batch normalization layer, as displayed in the figure. The node pooling layer coarsens the graph by pooling nodes of spatiotemporal proximity into one node.

A graph neural network, namely TactiGraph, with a stack of SplineConv layers and node pooling layers, is used for contact angle prediction. TactiGraph consists of SplineBlocks, node pooling layers, a final pooling layer, and a final multilayer perceptron (MLP). A graphical depiction of TactiGraph can be seen in Figure 3a. SplineBlocks consist of a SplineConv layer, an exponential linear unit (ELU), and a batch normalization layer, as depicted in Figure 3(I). Like any GNN layer, each SplineConv layer performs message passing between nodes to build a representation. What makes SplineConv appropriate for graphs constructed on events is that the messages encode the spatial geometry of the events. In particular, given an event $e_{i}=\left(x_{i},y_{i},t_{i},p_{i}\right)\in E_{j}$ with neighbors $N\left(e_{i}\right)\subset E_{j}$, SplineConv will build a representation as follows

(7) $$x_{e_{i}}^{\ell +1}=\sum_{k\;:\;e_{k}\in N\left(e_{i}\right)}W^{\ell }\left(x_{i}-x_{k},y_{i}-y_{k},t_{i}-t_{k}\right)x_{e_{k}}^{\ell }$$

where $W^{\ell }\in R^{n_{in}\times n_{out}}$ is a learnable function associated with the ℓth layer of the network, with $n_{in}$ and $n_{out}$ being the dimensionality of the input and output, respectively [64]. Thus what ends up being learned is a function of the relative positions of neighboring events which promotes learning the spatial geometry of the scene. To sum up, a $\mathrm{SplineBlock}(N,n_{in},n_{out})$ layer will take a graph *G* with *N* nodes, each of which has a node feature $x^{\ell }\in R^{n_{in}}$ and output updated node features $x^{\ell +1}\in R^{n_{in}}$.The node pooling layer reduces the number of nodes in the graph $G_{j}$ from $N_{i}$ to $N_{i+1}$ by first constructing a voxel grid over the volume $E_{j}$ then pooling all nodes within a voxel unit into one node; inheriting edges, if any, from the unpooled nodes. An example of pooling operation is shown in Figure 3(II) where a 3×3 voxels are used to pool the graph. For the layers before pooling, messages passed between events, ensuring that the initial low-level geometry is preserved. After pooling, higher-level nodes are created and message-passing occurs between high-level features. The final pooling layer will convert the variable size graph into a fixed-size vector by max pooling over a 4×4 voxel grid. A voxel grid is generated over the spatiotemporal volume E. Skip connections are also added by adding node features.

The final MLP layer of TactiGraph outputs a prediction estimating the contact angle roll and pitch. The predicted value is compared to the ground truth obtained using the UR10 as discussed in Section 2.1. Thus for every $(G_{j},{\mathrm{Roll}}_{j},{\mathrm{Pitch}}_{j})\in D$, TactiGraph predicts the contact angle $\left(\hat{{\mathrm{Roll}}_{j}},P{\hat{\mathrm{itch}}}_{j})\right)$. The error is then computed using the mean absolute error (MAE)

(8) $$\mathrm{MAE}=\frac{1}{n}\sum_{j=1}^{n}\left|X_{j}-{\hat{X}}_{j}\right|$$

where X∈{Roll,Pitch}. The values of roll and pitch evaluated used are the same as the data collection setup in Section 2.1 and ranges ∈$\left[-9^{\circ },9^{\circ }\right]$.

To select the optimal TactiGraph architecture, an automated search routine was developed using the training and evaluation set of $D_{\mathrm{LED}-\mathrm{on}}$ dataset. The automated search routine spanned several parameters that include the number of SplineConv layers, the number of node pooling layers, the number of skip layers, and the node-embedding dimension. Such a process is carried through using the asynchronous successive halving algorithm (AHSA) scheduler in the Ray Tune library [80]. This scheduler will compare the performance of the model at various checkpoints during training thus deciding whether to continue training the model or not. Specifically, the performance of all the attempted networks will be evaluated on the validation set of $D_{\mathrm{LED}-\mathrm{on}}$ in terms of the MAE metric. The AHSA scheduler is used with a grace period of 75 epochs, a reduction factor of 2, and maximum training epochs of 1000. This allows us to ablate over a large selection of the hyperparameters on TactiGraph. The range of these hyperparameters is shown in Table 1.

### 2.6. Training Setup

We use version 2.0.4 of the Pytorch Geometric library [81] to implement our models. Training is performed over 1000 epochs using the Adam optimizer [82] with an adaptive learning rate and default values as per Pytorch version 1.11.0 [83]. The learning rate starts at 0.001 but is reduced to 0.00001 when the validation loss plateaus. The training is carried out on a PC running Ubuntu 20.04, with an Intel i7-12700H CPU and an NVIDIA RTX 3080 Laptop GPU.

## 3. Results and Discussion

The proposed TactiGraph for predicting contact angles is tested both qualitatively and quantitatively in multiple cases, with and without internal illumination, to demonstrate its validity, robustness, and computational efficiency. In this section, we present our findings in benchmarking TactiGraph against other methods of contact angle prediction as well as other methods of processing event streams. We demonstrate the abilities of TactiGraph on N-VBTS with and without internal illumination. We conduct a computational analysis comparing TactiGraph on N-VBTS with conventional VBTS approaches.

### 3.1. Quantitative Evaluation

#### 3.1.1. Contact Angle Prediction Performance Evaluation

The best model from the ablation study is shown in Figure 3. The training results on both LED-on and LED-off datasets are shown in Table 2. We display TactiGraph’s mean absolute error (MAE), on the test dataset. The model is trained with and without applying the 1 px jitter augmentation on the training datasets. We note that applying the jittering augmentation strategy when training improves accuracy in the test dataset. The effect of jittering is amplified on the LED-off dataset. We argue that this is due to the noisy and sparse nature of event-based cameras in the LED-off environment [75,76]. Thus, exposing the model to jittered events makes the model more robust to the noisy test dataset. It is worth noting that jittering events by more than one pixel proved to be an ineffective strategy that gave worse results than not jittering. This might be due to the fact that the event-based camera used, the DAVIS 346c, is of a relatively low resolution. Thus, jittering by more than one pixel can cause a change in the true dynamics of the scene.

**Figure 4:**
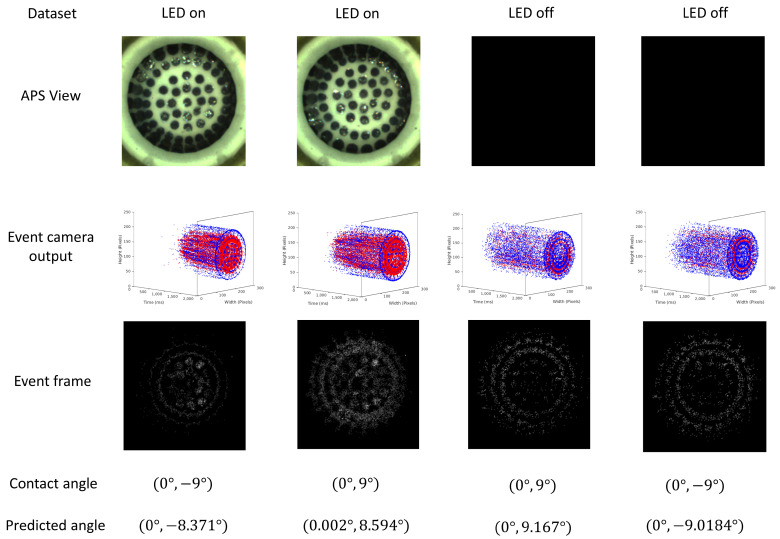
Samples of the dataset on the LED-on and LED-off datasets. The active pixel sensor (APS) views of a few samples from the datasets and their corresponding event streams E and event-frames *F*.

The translucence of the sensor tip, allowing some light to pass through, is a characteristic to consider. However, the working principle of event-based cameras addresses the generalizability issue when the LED is off. Event-based cameras primarily trigger events when there are moving edges or changes in the scene. In the case of N-VBTS, these moving edges are the markers contrasted against the background. This can be better seen in the APS (frame) view of the camera in Figure 4. It is important to note that event-based cameras do not capture color intensity information. This property enables TactiGraph to operate effectively under different external illumination conditions. With N-VBTS, the operation is more generalized to variation in the illumination compared to standard camera VBTSs that use semitransparent, transparent, or translucent skins such as those mentioned in [9,13,51,52], where external lighting conditions can significantly affect their output, thus altering the predictions made by downstream algorithms. This was discussed in more detail in Section 1.2. It is worth mentioning that extreme lighting conditions, such as complete darkness, are not within the scope of this work and may not be directly relevant to TactiGraph’s performance.

#### 3.1.2. Robustness of TactiGraph

**Figure 5:**
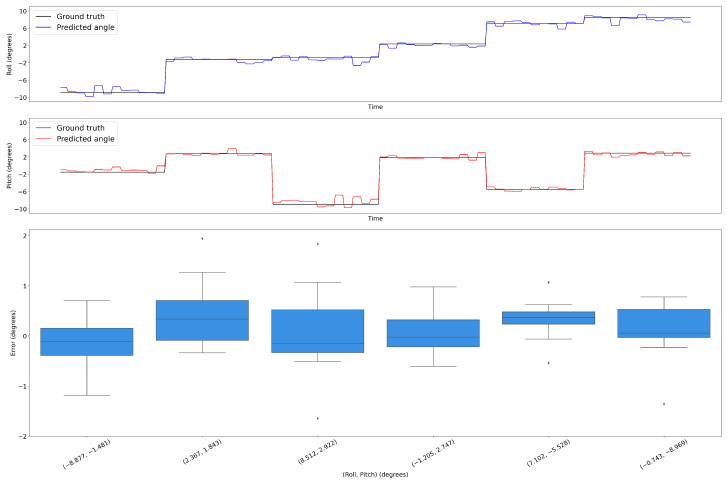
Illustrates the repeated contact made by the sensor at different roll and pitch values which are shown in the x-axis of Figure (**c**). The predicted values generated by TactiGraph are overlaid on the corresponding ground-truth values. Specifically, Figure (**a**) depicts the roll values, while Figure (**b**) shows the pitch values. The ground-truth values are represented in black, and the predicted values are superimposed on top of them. In addition, for each of the six possible combinations of contact angles, the boxplots in Figure (**c**) show the error in the estimated values.

Several experiments were conducted to evaluate the robustness of TactiGraph. In these experiments, the sensor was commanded to make contact at six different angles, with each angle being repeated 12 times. The experiments aimed to determine if TactiGraph’s contact angle predictions were consistent across the 12 trials. Figure 5 displays the results of the experiments, with the x-axis showing the six angles. The roll and pitch angles predicted by TactiGraph are imposed onto the true roll and pitch values in Figure 5a,b. The results demonstrate that TactiGraph consistently predicted angles that were very close to the true contact angle, indicating its robustness. The boxplots in the figure provide further evidence of TactiGraph’s robustness, with the errors mostly being within $\pm 1^{\circ }$ of the true value for all angles and always within $\pm 2^{\circ }$ of the ground-truth angle.

#### 3.1.3. Benchmark Results

The only contact angle estimation approach in the literature that uses neuromorphic vision-based tactile sensing is the work of MacDonald et al. [50]. However, unlike our work, MacDonald et al. estimate the contact angle with an edge rather than a flat surface; thus, these results are not directly comparable. They build an embedding using a spiking neural network in an unsupervised fashion, which is coupled with a supervised KNN classifier. We also compare our results against other works using traditional vision-based tactile sensing approaches. The results are tabulated in Table 3. We split the results between N-VBTS methods and conventional VBTS methods. Given the relatively low dynamic range of conventional cameras, conventional VBTSs do not work without an internal source of illumination. While Halwani et al. [28] achieve better results, this is accomplished by utilizing a CNN operating on a conventional camera, which necessitates a source of illumination. However, their approach is susceptible to motion blur and incurs higher computational costs, as demonstrated in Section 3.3. The same applies to Tac-VGNN [35], which uses a GNN. However, their GNNs operate synchronously on graphs constructed using a conventional camera, where graph nodes are made of internal markers.

To validate the effectiveness of TactiGraph in processing asynchronous event streams, we also developed a CNN-based network that operates on synchronous event frames. Specifically, in this CNN model, the event stream is projected onto a 2D frame. To ensure a fair comparison, we utilized the same event volume $E_{j}$ from both the LED-on and LED-off datasets. For every event volume $E_{j}$, a graph $G_{j}$ and an event-frame $F_{j}$ are constructed using the same volume. The graph is fed to TactiGraph while the event-frame is fed into the CNN.

The following presents the construction of a grayscale event frame $F_{j}\in R^{346\times 260}$ from the corresponding to volume $E_{j}$:

(9) $${\left(F_{j}\right)}_{ab}=\sum_{e_{i}\in E_{j}}p_{i}\delta \left(x_{i}=a\right)\delta \left(y_{i}=b\right),$$

where δ is the Dirac delta function. Sample event-frames are shown in Figure 4 where the temporal information of event streams is lost.

To determine the most suitable architecture for the CNN model operating on event frames, we conducted an ablation analysis. Inspired by the CNN structure proposed by Halwani et al. [28] for VBTS contact angle prediction on RGB images, we examined the network’s performance under different configurations, including variations in the number of channels in the 2nd, 3rd, and 4th convolutional layers, the number of convolutional layers, and the number and sizes of fully connected layers.

Similar to the search routine employed for TactiGraph (Section 2.5), we utilized the AHSA scheduler from the Ray Tune library [80] for the ablation process. Specifically, the performance of all the attempted CNN models was evaluated on the validation set of $D_{\mathrm{LED}-\mathrm{on}}$ using the mean absolute error (MAE) metric. The range of hyperparameters considered and the optimal values for the CNN architecture are presented in Table 4.

Table 5 reports the benchmark results of the best CNN model in comparison with TactiGraph. TactiGraph demonstrates superior contact angle estimation compared to the optimal CNN architecture when event-frames constructed from the same $E_{j}$ are considered. It is important to highlight that TactiGraph preserves the temporal feature of the event stream through its asynchronous operation for exploiting the spatiotemporal correlations between events. This plays a vital role in the overall performance of contact angle detection. This is evident in the improved performance results achieved by TactiGraph compared to the CNN model on event-frames, as observed in both LED-on and LED-off scenarios.

### 3.2. Qualitative Evaluation

#### 3.2.1. Sample Cases and Their Predicted Output

Figure 4 depicts a selection of samples from both illumination scenarios datasets, the LED-on and LED-off cases. Moreover, the images depict the APS view of two opposing contact cases, where the markers are visible when the LED is turned on, but not visible in the absence of illumination. This presents a challenge in estimating the deformation of the sensor and predicting the contact angle. It is worth noting that increasing the camera’s exposure time improves marker visibility, but at the expense of introducing motion blur, as observed in our experiments. On the other hand, event-based cameras do not suffer from this issue, and the 3D and 2D projections of the event streams associated with the contact cases clearly capture the motion of the markers caused by the deforming sensor as shown in Figure 4. Despite the absence of illumination, the event cameras’ high dynamic range and temporal resolution enable the generation of events, albeit only when there is motion. Furthermore, the temporal information contained in the sub 1 ms resolution events is essential, as demonstrated in Table 5. Importantly, when events are projected onto 2D images to form event-frames, significant properties of the event stream, such as temporal information, is lost. As a result, the observation of motion dynamics in the captured scene can be impeded, as illustrated in Figure 4.

#### 3.2.2. Visualizing TactiGraph’s Embedding Space

**Figure 6:**
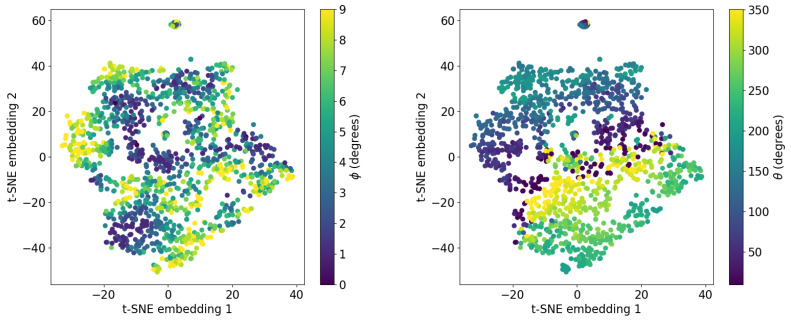
*t*-SNE embeddings of the activations of TactiGraph’s last layer before the MLP. Each point is the embedding of one graph $G_{j}$ corresponding to jth contact case. **Left**: colored by the ϕ angle of the contact case. **Right**: colored by the θ angle of the contact case.

To obtain a better understanding of what TactiGraph has learned, we visualize the node embedding generated for each sample in the dataset. This is performed by saving the values obtained from the last node pooling layer, right before the fully connected layer, during a forward pass on the trained model. These values live in a high-dimensional space. To this end, we use the *t*-distributed stochastic neighbor embedding (*t*-SNE) algorithm [84]. *t*-SNE, a manifold learning technique, nonlinearly projects high-dimensional data onto a low-dimensional space which is more tangible for visualization.The results of the *t*-SNE visualization are shown in Figure 6. Each point in the scatter plot represents a contact case *j* associated with $\left(G_{j},{\mathrm{Roll}}_{j},{\mathrm{Pitch}}_{j}\right)\in D_{\mathrm{LED}-\mathrm{on}}$. The points are colored according to the angles of contact θ and ϕ. Even though TactiGraph was trained on roll and pitch representation, what we see in these plots is that TactiGraph has learned to embed similar contact angles θ and ϕ next to each other. Looking at how different values of ϕ and θ vary in the embedding space, we see that the model has learned an embedding that emphasizes variance in ϕ. This is due to the fact that ϕ varies more than θ. The clearly visible gradients in these plots confirm that TactiGraph has actually done a good job of learning the dynamics of the problem.

#### 3.2.3. Node Embeddings

**Figure 7:**
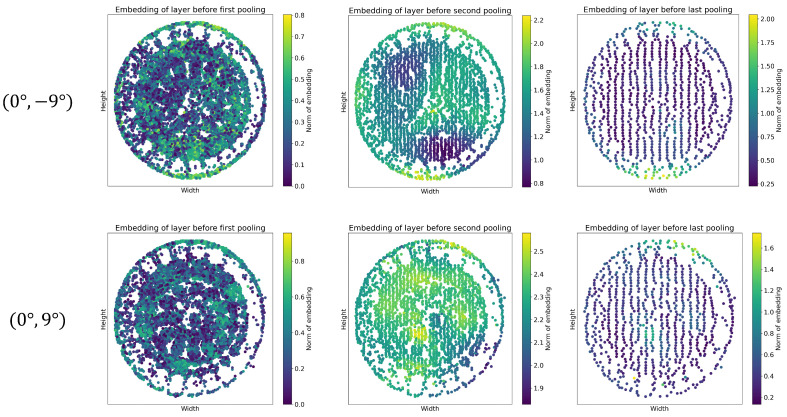
Node embeddings generated by forward passes through TactiGraph. The first row is a contact case at angle $\left(0^{\circ },-9^{\circ }\right)$ while the second row is angle $\left(0^{\circ },9^{\circ }\right)$. Each column shows the norm of the node features before every pooling layer of TactiGraph. The first column shows the embedding generated by layer 3, the second column shows the embedding from layer 7, and the last column shows the embeddings from layer 12 of TactiGraph. The embeddings are generated using Equation (10). In column one, each point in the plot corresponds to an event.

On every layer of a graph of a neural network, including TactiGraph, nodes update their node features via aggregating information from their neighbors. Thus in a GNN with only message-passing layers, such as SplineConv, the receptive field of every node is limited by the number of message-passing layers in the GNN. Similar to pooling layers in a CNN, pooling layers in GNNs allow nodes to expand their receptive field. Our ablation study showed that three pooling layers were optimal for TactiGraph. The first few layers of SplineConv happen on the event levels where nodes are events themselves. Thus, before the first pooling layer, low-level message-passing between events occurs. With every pooling layer, message-passing is at a higher level than before. We visualize the node features learned at each level of message-passing in Figure 7. We look at the norm of the embeddings generated in the layers pre-pooling in a forward pass of two samples, the same first samples from Figure 4. The norm embedding of a node $e_{i}$ in a graph $G_{j}\in D$ on layer *ℓ* is given by

(10) $$\left|\right|x_{e_{i}}^{\ell }\left|\right|=\sqrt{\sum_{m=1}^{n_{out}}{\left(x_{e_{i}}^{\ell }\right)}_{m}^{2}}$$

where $n_{out}$ is the dimensionality of layer *ℓ* and *m* iterates over $\left\{1,2,\ldots ,n_{out}\right\}$.The norm of the embedding in TactiGraph is computed for each node individually. Before the last layer of TactiGraph is a max pooling layer, which outputs nodes with higher norm values. These nodes play a significant role in angle predictions. Figure 7 shows the visualization of node norm embedding for two angle predictions. Nodes with higher importance, indicated by higher norm values, are concentrated around specific regions. This mapping reflects the direction of the predicted contact angles in TactiGraph, as demonstrated in both contact cases ($\left(0^{\circ },-9^{\circ }\right)$ and ($0^{\circ },9^{\circ }$)). More specifically, before the first layer, nothing much seems to happen. This is expected, as event-based cameras are known to be noisy, hence it is expected that the first few instances of message-passing will be noisy. The second pre-pooling embedding is more informative; TactiGraph correctly highlights the correct directions but is still not sure. Finally, on the final layer, the model correctly highlights the direction of the contact angle. An accurate prediction is then made by the linear layer after pooling, as tabulated in Figure 4.

### 3.3. Inference Time Analysis

Given a live event stream S that started running at time $t_{0}$, in other words, for every (x,y,t,p)∈S, we have $t_{0}\leq t\leq t_{c}$, where $t_{c}$ is the current time. TactiGraph operates on a graph constructed from a sliding window.

(11) $$W=\{\left(x,y,t,p\right)\in S\;|\;t_{c}-\Delta T\leq t\leq t_{c}\}$$

As events asynchronously enter and exit W, the graph is updated accordingly and TactiGraph acts on it. Instead of having a GNN rerun the whole forward pass as events slide in and out of W, Schaefer et al. [68] propose AEGNN, a method by which redundant computations are not repeated. By looking at the neighborhoods of incoming and outcoming events, AEGNN is able to asynchronously determine which computations need to be recomputed. We modify TactiGraph to utilize the same mechanism as AEGNN. With these optimizations in mind, a prediction using TactiGraph consists of two steps: graph construction and the forward pass. In the worst-case scenario where the whole scene changes, the graph construction step takes an average of 34.5 ms. In addition, in the worst-case scenario, the forward pass takes an average of 58.1 ms. These results were obtained on the same hardware mentioned above in Section 2.6.

The combination of N-VBTS and TactiGraph is computationally much cheaper than the CNN and VBTS of Halwani et al. [28]. We validate this by looking at the total computing time taken by both methods in processing the same contact cases. We record a dataset 20 s long containing five contact cases. We run the CNN model from [28] on the active pixel sensor stream of the same DAVIS 346c used in this work. The total computing time the CNN takes to process this stream is 3.93 s. TactiGraph operating on the event stream, on the other hand, took only 0.22 s, 5.5% of the CNN computing time. This is attributed to the redundant output the VBTS gives, which leads to redundant computations by the CNN. Therefore, TactiGraph operating on N-VBTS streams is much faster than the CNN model operating on VBTS streams from [28].

The quick and accurate detection of tactile normality is crucial for maintaining product and tool quality in automated machining. It enables prompt feedback and facilitates immediate corrective actions, such as adjusting robot arm parameters, to ensure effective operation in downstream tasks. Swift identification of normal tactile angles contributes to overall productivity by optimizing operational speeds and minimizing unplanned downtime, thereby enhancing overall equipment effectiveness.

### 3.4. Future Work

Our neuromorphic vision-based tactile sensor has shown remarkable performance in contact angle prediction. Therefore, the TactiGraph capabilities can be extended further to perform other tactile sensing applications such as force sensing, texture recognition, and object identification in parallel. We plan on also including a recurrent or attentional mechanism in TactiGraph. This will give TactiGraph the generalization ability to operate on multiple tasks. It is also worth noting that the forward pass in TactiGraph can be further improved by replacing SplineConvs with a corresponding look-up table as proposed in [70], which claims a 3.7-fold reduction in inference time.

## 4. Conclusions

We introduced a neuromorphic vision-based tactile sensor (N-VBTS) that is able to run at a faster rate than the traditional vision-based tactile sensor (VBTS). N-VBTS mitigates the exposure time latency in conventional VBTS by utilizing an asynchronous and continuous-in-time event-based camera.We developed TactiGraph, a graph neural network, to operate on the raw asynchronous event stream exploiting the spatiotemporal correlations between events, hence making use of the low latency perception provided by N-VBTS. Notably, TactiGraph is utilized to predict the contact angle of the sensor and achieves an error of $0.62^{\circ }$ degrees. We demonstrated the effectiveness of the proposed N-VBTS in terms of efficacy and accuracy compared to VBTS. In particular, N-VBTS was capable of functioning without internal illumination hence leading to a reduction in long-term instrumentation and maintenance requirements. When tested on the same scenario, N-VBTS requires only 5.5% of the computing time needed by VBTS. 

## Figures and Tables

**Table 1:** Hyperparameter search for SplineConv graph neural network.

Hyperparameter	Hyperparameter Range	Optimal Value
Number of SplineConv layers	{6, 7, 8, 9, 10, 11}	10
Number of channels in layers	{8, 16, 32, 64, 128}	(8, 8, 16, 16, 16, 16, 32, 32, 32, 32)
Number of pooling layers	{1, 2, 3}	3
Number of skip connections	{0, 1, 2, 3, 4}	3

**Table 2:** MAE of training TactiGraph on the dataset with the LED on and the dataset with the LED off. We compare results before and after adding jittering augmentation.

Dataset Used	MAE before Jittering	MAE after Jittering
$D_{\mathrm{LED}-\mathrm{on}}$	$0.65^{\circ }$	0.63${}^{\circ }$
$D_{\mathrm{LED}-\mathrm{off}}$	$0.81^{\circ }$	0.71${}^{\circ }$

**Table 3:** MAE of TactiGraph compared to VBTS methods from the literature.

	Neuromorphic VBTS		VBTS	
Internal illumination ${}^{‡}$	TactiGraph	MacDonald et al. [50] ${}^{†}$	Halwani et al. [28]	Tac-VGNN [35]
With illumination	$0.63^{\circ }$	$4.44^{\circ }$	$0.13^{\circ }$	$1.02^{\circ }$
Without illumination	$0.71^{\circ }$	-	-	-

${}^{†}$ Contact is not made against a flat surface. ${}^{‡}$ For TactiGraph results, the datasets used for different illumination conditions are $D_{\mathrm{LED}-\mathrm{on}}$ and $D_{\mathrm{LED}-\mathrm{off}}$ as described in Section 2.

**Table 4:** Hyperparameter search for a convolutional neural network on event-frames. The architecture with the lowest error is used for comparison against TactiGraph.

Hyperparameter	Hyperparameter Range	Optimal Value
Number of convolutional layers	{3,4,5,6,7}	6
Number of channels in layers	{16,32,64,128,256}	(32, 32, 32, 128, 128, 256)
Number of dense layers	{2,3,4,5}	4

**Table 5:** MAE of TactiGraph compared to CNN on event-frame.

Dataset Used	TactiGraph	CNN on Event-Frame
$D_{\mathrm{LED}-\mathrm{on}}$	0.63${}^{\circ }$	0.79${}^{\circ }$
$D_{\mathrm{LED}-\mathrm{off}}$	0.71${}^{\circ }$	$0.80^{\circ }$

## Data Availability

The data presented in this study are openly available on GitHub at https://github.com/HussainMSajwani/tactile.

## Abbreviations

The following abbreviations are used in this manuscript:

VBTS	Vision-based tactile sensing
N-VBTS	Neuromorphic vision-based tactile sensing
APS	Active pixel sensor
LED	Light-emitting diode
GNN	Graph neural network
CNN	Convolutional neural network
SNN	Spiking neural network
MAE	Mean absolute error
kNN	*k*-nearest neighbors
*t*-SNE	*t*-distributed stochastic neighbor embedding
